# Application of LSTM Network to Improve Indoor Positioning Accuracy

**DOI:** 10.3390/s20205824

**Published:** 2020-10-15

**Authors:** Dongqi Gao, Xiangye Zeng, Jingyi Wang, Yanmang Su

**Affiliations:** 1 Hebei Key Laboratory of Advanced Laser Technology and Equipment, School of Electronics and Information Engineering, Hebei University of Technology, Tianjin 300401, China; 201831903007@stu.hebut.edu.cn; 2 Tianjin Key Laboratory of Electronic Materials and Devices, School of Electronics and Information Engineering, Hebei University of Technology, Tianjin 300401, China; wangjingyi@hebut.edu.cn (J.W.); sym@hebut.edu.cn (Y.S.)

**Keywords:** indoor positioning, ultra-wide band (UWB), LSTM, ranging error prediction, least squares, deep learning

## Abstract

Various indoor positioning methods have been developed to solve the “last mile on Earth”. Ultra-wideband positioning technology stands out among all indoor positioning methods due to its unique communication mechanism and has a broad application prospect. Under non-line-of-sight (NLOS) conditions, the accuracy of this positioning method is greatly affected. Unlike traditional inspection and rejection of NLOS signals, all base stations are involved in positioning to improve positioning accuracy. In this paper, a Long Short-Term Memory (LSTM) network is used while maximizing the use of positioning equipment. The LSTM network is applied to process the raw Channel Impulse Response (CIR) to calculate the ranging error, and combined with the improved positioning algorithm to improve the positioning accuracy. It has been verified that the accuracy of the predicted ranging error is up to centimeter level. Using this prediction for the positioning algorithm, the average positioning accuracy improved by about 62%.

## 1. Introduction

By locating people and assets outside of GPS [1] coverage and accelerating business processes such as the Internet of Things (IoT) and smart buildings, wireless positioning technology has a broad market prospect and demand in today’s era.

Since human beings spend more than 50 percent of their time in indoor environments, location-based services (LBS) in indoor environments are of increasing interest. A large number of emerging technologies and technology-related algorithms have been developed for indoor positioning, and the most important ones are ZigBee, radio frequency identification (RFID) [2], fingerprinting-based methods [3], infrared positioning [4], Bluetooth and ultra-wide band (UWB). Of all these technologies, the UWB technique has become the most promising and important means of solving indoor positioning problems because of its extremely high time-domain resolution and strong penetration capabilities.

Theoretically, UWB positioning systems could perform centimeter- or even millimeter-level positioning in line-of-sight (LOS) conditions. However, in the actual indoor positioning environment, the signal is blocked by people, and walls and other obstacles in the process of propagation and reflection, refraction, diffraction and other phenomena. The transmit signal reaches the receiving end with the multipath effect, and the propagation signal has different degrees of time delay, which is the main source of indoor positioning error. This phenomenon is also known as non-line-of-sight (NLOS) interference. To solve the problem of NLOS influence, much of the previous works revolved around extracting channel features for NLOS identification and correction. For example, [5] proposes a method for classifying propagation conditions between tags and anchors into three categories based on the first path component power, combined with the EKF algorithm and classification results corrected in the position measurement process. Another example is [6], which analyzes the waveform based on the proposed seven features, and uses a support vector machine (SVM) to distinguish LOS/NLOS propagation, and further reduces the ranging error under NLOS conditions. In [7], the strength of the received signal and other statistical properties of the range results to implement LOS and NLOS classification using an SVM classifier are characterized.

Since Channel Impulse Response (CIR) reflects the path a signal travels from the transmitter to the receiver, in the application of UWB positioning systems, the channel impulse response is usually measured. In fact, the ranging error depends on the physical environment and the specific obstacles in the environment. If the objects encountered during the signal propagation are different, the CIR will also change [8,9]. Based on this fact, this paper analyzes the CIR signal to predict the ranging error without additional feature extraction.

Compared to the frequency of change of the target position at a fixed point or low speed travel and the dense signal transceiver frequency between anchor and target node, the change of CIR is a relatively “slow” process. This is the reason why we consider the use of neural networks with memory function in time correlation to explore the mapping between CIR and ranging error.

The relative position relationship between the target node and the anchor, which carries the spatial information between the target node and the anchor, affects channel impulse response. The Long Short-Term Memory (LSTM) network used to process CIR signals has a memory function. While autonomously remembering CIR signals, it also remembers the distribution of objects in the positioning space.

The main contributions of this paper include:

1. No need for LOS/NLOS distinction, processing channel impulse responses predicts ranging error using LSTM Networks with memory functions. The predicted results are used to build a weight matrix to improve the accuracy of positioning.
2. Since the correlation between the CIR signals is captured, the training effect shows that the accuracy of the distance measurement error predicted by the LSTM network reaches the centimeter level.
3. The ranging error predicted by the LSTM network is used for positioning correction. The results show that the worse the positioning conditions, the greater the error correction value. In an environment with good positioning conditions, the corrected positioning results can meet most positioning requirements.

## 2. Materials and Methods

UWB wireless positioning systems include mobile station-based, wireless communication network-based and hybrid positioning methods. In practical applications, wireless communication network-based positioning methods are mostly used. At present, common ranging technologies are based on time of arrival (TOA) [10,11], time difference of arrival (TDOA) [12,13], angle of arrival (AOA) and based on received signal strength (RSS). Among them, due to the excellent time resolution of UWB-, TOA- or TDOA-based ranging technology is more widely used, while RSS [14] and AOA [15] are more used as auxiliary means.

UWB positioning, as a range-based positioning technology, requires that the anchors whose relative positions are known be deployed in advance, and the distance between the anchors and the target node will be returned by the sensor. Therefore, the accuracy of the distance measurement directly affects the accuracy of the positioning results. Methods for estimating target node locations based on distance information can be roughly divided into two categories. One is based on optimization calculations, and the other is based on non-optimization algorithms. Algorithms that are not based on optimization usually have low accuracy, and the advantage is that the amount of calculation is small—for example, the Fang algorithm, which can estimate the position of the target node in a two-dimensional plane based on the information of three base stations, but the algorithm can and can only use the information of the three anchors, which makes the accuracy of the solution very limited. To include more anchors’ information in the solution, it is necessary to utilize an optimization-based algorithm. The most widely used and most promising numerical optimization algorithms usually establish the objective function based on the maximum likelihood criterion. For example, in the least square (LS) method used in this article, the optimization goal of the algorithm is to minimize the square error. Another example is maximum likelihood (ML) estimation, with the minimum error likelihood function values as the optimization objective.

In many applications of least squares calculation algorithms, such as the Chan [16] algorithm based on TDOA positioning [17], Friedlander algorithm and Taylor [16] series expansion method, the system measurement error is assumed to obey a Gaussian distribution with zero mean. However, due to the influence of NLOS errors in the actual environment, this premise does not hold. To solve this problem, the most common idea is to perform NLOS discrimination based on channel status and channel characteristics [11]. The features used for NLOS discrimination are derived from the extraction of channel statistical information, including received signal strength, skewness, kurtosis, root mean square (RMS) and average excess delay [18]. If it is determined that the channel is NLOS, discard or error correction is performed. Support vector machines (SVM) [6,19] and relevance vector machines (RVM) [20] play important roles in NLOS detection and elimination due to their powerful classification and regression capabilities. There are also some studies using CNN networks [21,22] to identify NLOS. The method of discarding the NLOS channel requires sufficient anchors, which causes a waste of resources. Therefore, it is more realistic to correct the ranging results.

As a function “black box”, neural network can continuously learn the mapping relationship between input and output according to different engineering needs to fit the expected result. The general neural network believes that the relationship between the inputs is discrete, while the RNN network can capture the connection between the front and back inputs. The signal receiving and sending frequency between the anchor and the target node far exceeds the frequency of the relative position change between the two, so we believe that there is a temporal correlation between the CIR signals which are intensively exchanged. As a variant of the RNN network, the LSTM network [23] can solve the problem of gradient explosion and gradient disappearance of ordinary RNN network through three gates set by neurons. To the best of our knowledge, there are no more studies using the LSTM network for ranging error prediction. The network training uses the open source data set [21], which is based on the standard UWB positioning sensor DW1000.

**Figure 1:**
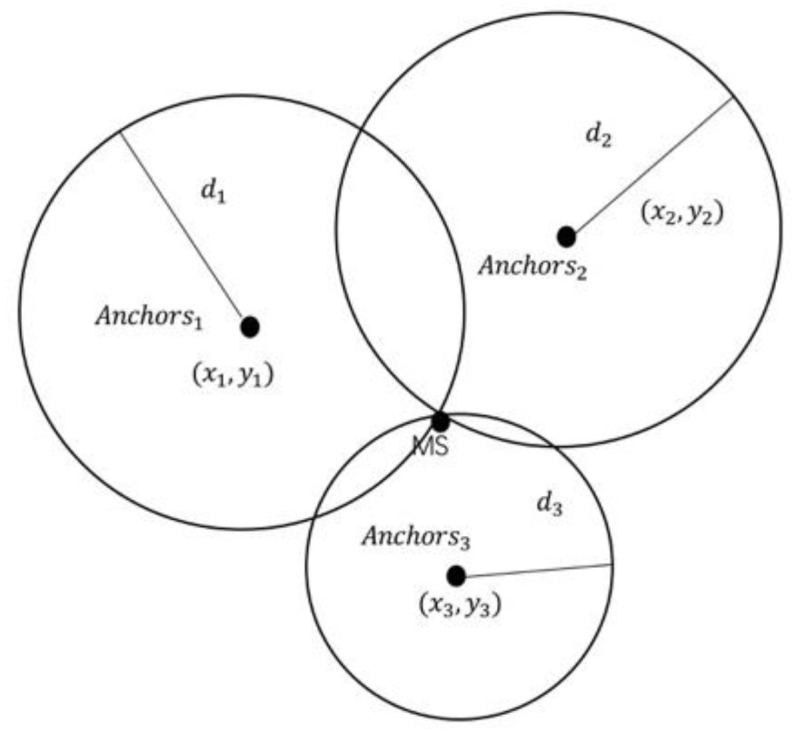
Trilateral positioning schematic.

The positioning method used by the DW1000 sensor is based on the TOA. TOA is a positioning method based on the time of arrival, or the so-called time-of-flight (TOF). By analogy, at least 3 anchors with known positions are required in the two-dimensional positioning. However, the trilateral positioning method (Figure 1) is an ideal model. In the real world, the three circles usually do not intersect at one point, and may even not intersect at all, resulting in no solution to the equation system. In the actual positioning process, we need to use algorithms to estimate the optimal solution. The least squares method and the weighted least squares method (Appendix C for details) are adopted in this paper.

## 3. Results

### 3.1. System Architecture

**Figure 2:**
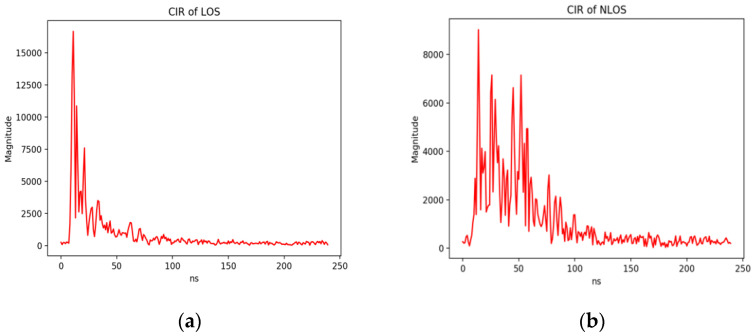
These figures show the difference of Channel Impulse Response (CIR) signals in line-of-sight (LOS) and non-line-of-sight (NLOS). (**a**) CIR of LOS; (**b**) CIR of NLOS.

Under LOS and NLOS conditions, the channel impulse response has different time domain performance. Figure 2 shows the typical CIR collected by the UWB system in an office environment, and the image very intuitively shows the difference of the CIR signal in the two cases. Under LOS conditions, CIR signal has a more prominent first path and a more concentrated energy distribution. Under NLOS conditions, CIR shows obvious multipath interference. Since the CIR of UWB has a short duration, the first path can be separated from other reflected or scattered paths. As far as the positioning scenarios involved in the data set are concerned, extracting the first path component of the CIR for network training can reduce data redundancy and speed up training.

**Figure 3:**
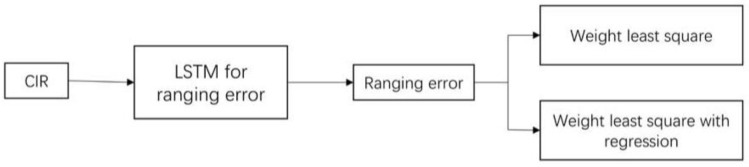
System architecture.

**Figure 4:**
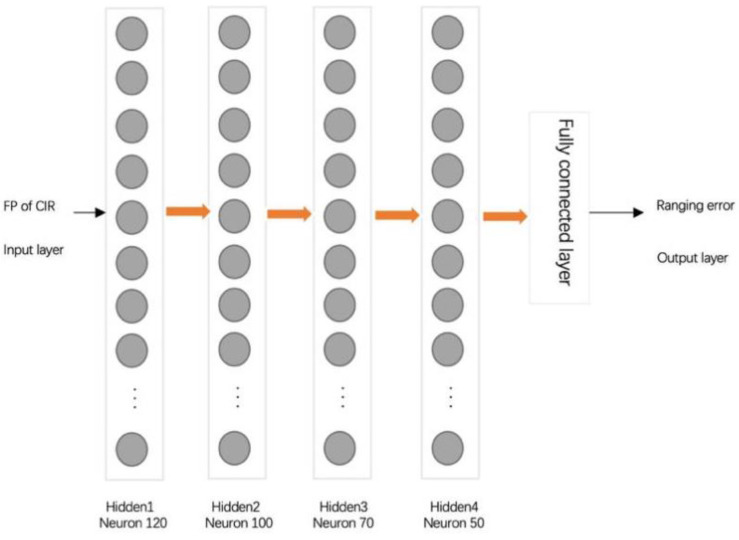
Long Short-Term Memory (LSTM) for ranging error.

The overall system architecture is shown in Figure 3, and the core part is the LSTM network (Figure 4) used for predicting ranging errors.

The public dataset we used is divided into two parts. The first part was recorded in two adjacent office rooms with the connecting hallway parallel to both the offices. The second part was recorded in a different office environment with multiple rooms included. Two tags were placed at fixed positions in each part, and different numbers of anchor positions were recorded. Table 1 shows the detailed data structure, including the number of anchor positions (APs) and the number of LOS and NLOS available for each tag: 100 samples for each anchor position.

The data set records the coordinates of the tags, the real position of the anchors and the measured position and the absolute value of CIR: 1016 points with 1 nanosecond and the index of first path which can be used for accessing the first path. In the experiment, the network processed the first 250 points of the first path indexed by the index.

According to the ratio of 60%, 20% and 20%, the data set is divided into three parts, which were used as a training set, validation set and test set, respectively.

### 3.2. LSTM for Ranging Error

There are four hidden layers in the ranging error prediction network, and the number of neurons in each hidden layer is different. Unlike ordinary neurons, which are usually equivalent to a single function, LSTM neurons have a more complex internal structure (Appendix A for details). The realization of the LSTM network memory function relies on three gated switches: input gate, output gate and forget gate. The splicing vector formed by the current input $x_{t}\;$ and the previous state $h_{t-1}$ is used as the input of the three gates. The input is converted to a value between 0 and 1 under the sigmoid activation function, thereby forming the switching control state of the three doors.

The CIR signal input to the network is a segment of continuously varying values, with small changes between the front and back inputs over a short period of time. However, when there is a sudden and large change in the relative position between the base station and the target, such as from the LOS state to the NLOS state, there may be a large difference between the front and back inputs. The CIR signal input to the network is a continuously changing value. During the data collection process, the sampling value may drift due to environmental noise interference. Since the BPTT algorithm used in the training process of the LSTM model is similar to the classical backpropagation algorithm [23], in order to prevent the wrong prediction results caused by erroneous data fluctuations from being superimposed continuously, the loss function of the network was set to Log-Cosh. This is because the Log-Cosh function is similar to the root mean square error function, but it is not easily affected by abnormal inputs.

There are many optimization algorithms based on gradients, such as stochastic gradient descent, AdaGrad and RMSProp. The optimizer selected in this paper is the adaptive momentum estimation (Adam) algorithm. This algorithm combines the advantages of AdaGrad and RMSProp, can calculate the adaptive learning rate of different parameters and occupies a small amount of storage resources and is an effective gradient-based stochastic optimization algorithm. In order to refine the learning process and find the extreme points in the true sense, the network automatically adjusts the learning rate (LR) to 0.8*OLD_LR after every 50 epochs. In order to prevent over-fitting, each hidden layer and fully connected layer randomly dropout 25% of the data.

**Figure 5:**
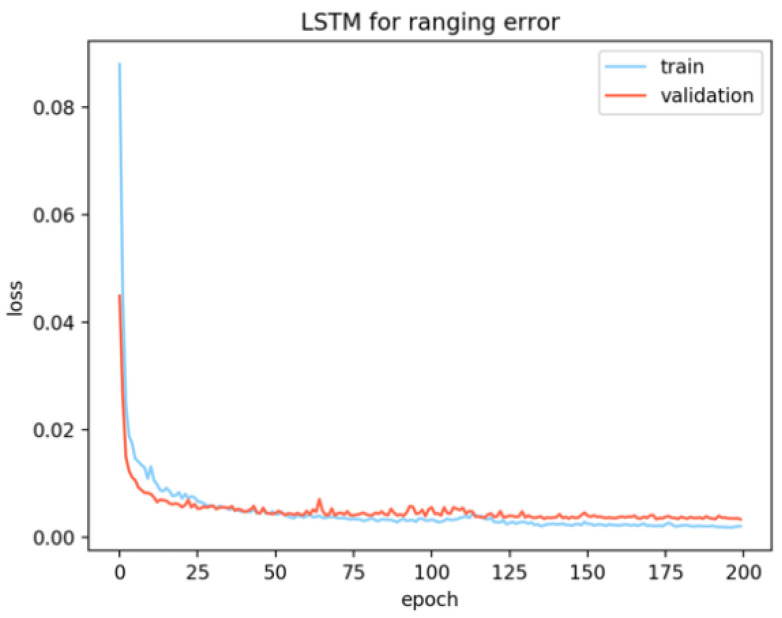
Model loss.

After 200 epochs, the network’s optimization result of the loss function tends to be stable (Figure 5), which means that the network has found the mapping relationship between the input sequence and the expected output. After training, save the network structure and all parameter values, and input the test set data into the network to predict the corresponding ranging error.

Record the model’s predicted value of the ranging error as ${\hat{e}}_{i}$, and the actual ranging error as $e_{i}$, and calculate the average difference between all predicted results and the true value:

(1) $$E_{m}\;=\frac{1}{n}\overset{n}{\underset{i=1}{\sum }}\left|\left|{\hat{e}}_{i}\;-\;e_{i}\right|\right|$$

The data set records the measurement results of different indoor environments in two parts. The positioning environment of the first part is offices and corridors, and the types of indoor spaces covered in the second part are more diverse. The network was used to predict the ranging error of the two parts of the data. The average difference between the predicted value and the actual value is shown in Table 2. The result shows that the model based on the CIR signal predicts the ranging error in accuracy of centimeter level.

### 3.3. Positioning Optimization Results

In the process of solving the equation system based on the trilateral ranging model, the linear variable R is introduced, then the target position can be estimated by least squares as:

(2) $$\hat{\theta }\;=\;{\left(H^{T}H\right)}^{-1}H^{T}x$$

(3) $$R\;=\;x^{2}\;+\;y^{2}$$

where $\hat{\theta }$ denotes the location information of the target node, $\;\hat{\theta }\;=\;{\left[x\;y\;R\right]}^{T}$ (Appendix B for details).

The default premise of using the least square method in UWB positioning is that the variance of the ranging error remains unchanged. However, in the actual positioning process, the ranging error under NLOS conditions is greater than that of LOS, and increases with the severity of NLOS. So, by simply using least square estimation, the positioning results are very poor.

Calculation of the weight $w_{i}$ of the individual range results using the sensor-estimated distance $d_{i}$ and the ranging error $\epsilon_{i}$ is adopted in this paper. In this way, the relative position relationship with long distance and large ranging error will be given lower weight, while the relationship with short distance and small ranging error will be considered more reliable.

(4) $$w_{i}\;=\;\frac{1}{{\left(d_{i}\epsilon_{i}\right)}^{2}}$$

The weight matrix is formed based on the above weight calculation participates in the position calculation of the target node, and the weighted least squares algorithm can be expressed as

(5) $${\hat{\theta }}_{WLS}\;=\;{\left(H^{T}WH\right)}^{-1}H^{T}Wx$$

Another compensation method is to modify the specific value of the x matrix in the ranging distance item on the basis of weighted least squares, and use the ranging distance minus the ranging error to form the ranging result to calculate the location. That is, the term ${\hat{d}}_{i}^{2}-\;x_{i\;}^{2}-\;y_{i}^{2}$ of the x is revised to ${\left({\hat{d}}_{i}\;-\;\epsilon_{i}\right)}^{2}-\;x_{i\;}^{2}-\;y_{i}^{2}$.

As a positioning method based on the distance measurement, the accuracy of the distance measurements is particularly important. In the actual positioning process, due to problems such as multipath interference and NLOS, the theoretical minimum number of anchors involved in positioning cannot reach the expected positioning results, so the discussion of two-dimensional positioning in this article starts with four anchors.

In order to better fit the real positioning conditions, each set of experiments designed in this paper ensures that at least two anchors are in NLOS conditions. The anchors participating in the positioning are randomly selected, and for each tag location, this random process is repeated 100 times. The ranging error predicted by the LSTM network is used in the position estimation algorithm to test the contribution of the network in positioning correction.

**Figure 6:**
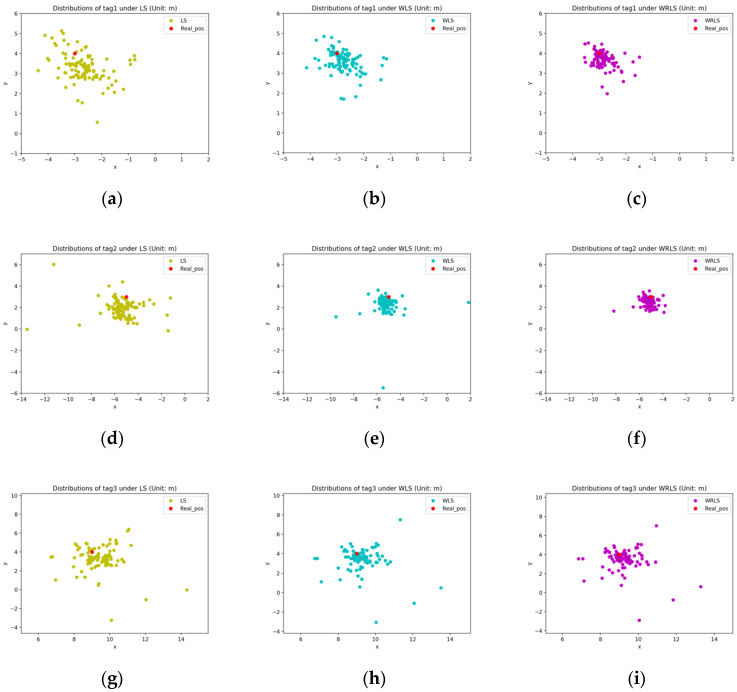

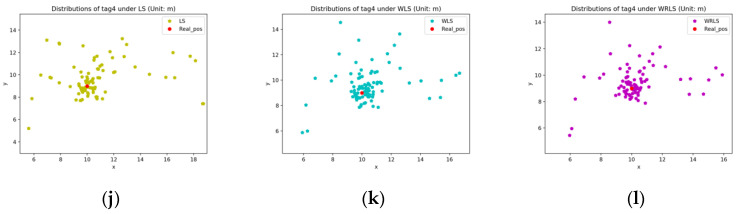
Distribution of target node locations under different algorithms. (**a**) The location distribution of tag1 under least square estimation; (**b**) the location distribution of tag1 under weighted least squares; (**c**) the location distribution of tag1 under weighted least square estimation with regression; (**d**) the location distribution of tag2 under least square estimation; (**e**) the location distribution of tag2 under weighted least squares; (**f**) the location distribution of tag2 under weighted least square estimation with regression; (**g**) the location distribution of tag3 under least square estimation; (**h**) the location distribution of tag3 under weighted least squares; (**i**) the location distribution of tag3 under weighted least square estimation with regression; (**j**) the location distribution of tag4 under least square estimation; (**k**) the location distribution of tag4 under weighted least squares; (**l**) the location distribution of tag4 under weighted least square estimation with regression.

In the experiment of four anchors participating in target node positioning, we recorded all the position solution results for all four tags, and the distribution is shown in Figure 6. It can be seen intuitively from the distribution map that the distribution area of the positioning result after error correction is more concentrated and the distribution shape is more convergent. Most of the points closely surround the actual position, and for some positioning results with large offsets, they are closer to the true value after error correction.

Select the positioning scenario of tag4 for further experimental verification, increase the number of anchors and keep at least two anchors participating in the positioning under NLOS conditions each time. In order to verify that the LSTM network’s prediction of the ranging error is equally effective under LOS and NLOS conditions, and to verify the rationality of the algorithm weight setting and the reliability of the compensation effect, in the experiment of eight anchors participating in positioning, two NLOS anchors and two LOS anchors are set, and the remaining four anchors are randomly selected. Table 3, respectively, counts the average difference between the positioning results of the three position estimation algorithms and the true value under the condition that 4 to 8 anchors participate in positioning.

**Figure 7:**
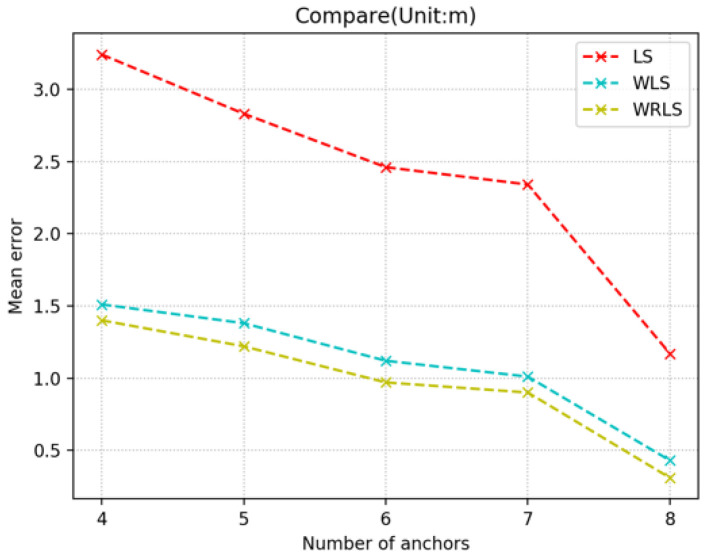
Comparison of solving results for each algorithm.

The positioning accuracy increases with the increase in the number of anchors involved in positioning (Figure 7), especially when eight anchors are involved in positioning; as long as two anchors are in LOS conditions, the positioning accuracy can be limited within 0.5 m by correction. The optimal average is as high as 31 cm. The experimental results of each group show that the weight matrix set by the prediction results of the network greatly improves the positioning accuracy of the weighted least squares method compared with the least squares method, and the regression weighting method makes a more detailed calibration on the basis of the weighted least squares method.

Table 4 shows the improvement in positioning accuracy of the two correction algorithms compared with the least square method. It can be seen that the fewer the number of anchors involved in positioning and the worse the conditions, the greater the improvement in positioning accuracy of the two compensation algorithms. In the experiment where four anchors participated in the positioning, the maximum correction value was as high as 1.84 m. In the experiment in which eight anchors were involved in positioning with relatively good conditions, through the range error prediction and compensation calculation, the difference between the estimated position and the actual position was reduced to within 0.5 m, which can meet the needs of most positioning applications.

In actual positioning, the target node and the anchor may be in any relative position, and the geometric shapes presented by the anchors participating in the positioning may also be non-convex shapes. However, as long as a high-precision prediction of the ranging error is made and used for algorithm compensation, it is possible to achieve the maximum positioning accuracy under limited conditions and resources.

## 4. Conclusions

In UWB-based indoor positioning technology, the frequency of signal transmission and reception between anchors and target nodes is higher than the frequency of relative position changes in most cases. According to this characteristic, this paper proposes a method of using an LSTM network to predict the ranging error between the anchor and the target. The LSTM network built and trained in this paper can predict the ranging error based on the input CIR signal, and the accuracy is up to centimeter level, using the predicted ranging error to adjust the weight of a single positioning information and correct the distance. After verifying that the LSTM network can predict the ranging error with higher accuracy, we design five sets of experiments with different number of anchors involved in positioning. The experimental conditions designed in this paper are more in line with the actual positioning environment, and at least two anchors in each group of experiments are in the NLOS state. Most of the previous experiments minimize the influence of NLOS by eliminating the anchors under NLOS conditions or by iteratively optimizing the combination of positioning anchors. This is why we did not make a quantitative comparison with prior work. Experimental results show that the worse the positioning conditions, the higher the difficulty, and the more significant the effect of algorithm correction combined with network prediction results. When the positioning conditions are better, the positioning accuracy can meet the requirements of most applications.

## Figures and Tables

**Table 1:** Data structure.

Part1	APs	LOS/NLOS	Part2	APs	LOS/NLOS
Tag1	48	17/31	Tag3	35	13/22
Tag2	51	15/36	Tag4	36	6/30

**Table 2:** Average difference between predicted and actual values (Unit: m).

Part 1	$E_{m}$	Part 2	$E_{m}$
Tag1	0.032	Tag3	0.022
Tag2	0.039	Tag4	0.018

**Table 3:** Average difference between real and measured positions (m).

Anchors	4	5	6	7	8
LS	3.24	2.83	2.46	2.34	1.17
WLS	1.51	1.38	1.12	1.01	0.43
WRLS	1.40	1.22	0.97	0.90	0.31

**Table 4:** Accuracy enhancement.

Anchors	WLS (m)	WRLS (m)
4	1.73	1.84
5	1.45	1.61
6	1.34	1.49
7	1.33	1.44
8	0.74	0.86

**Figure A1:**
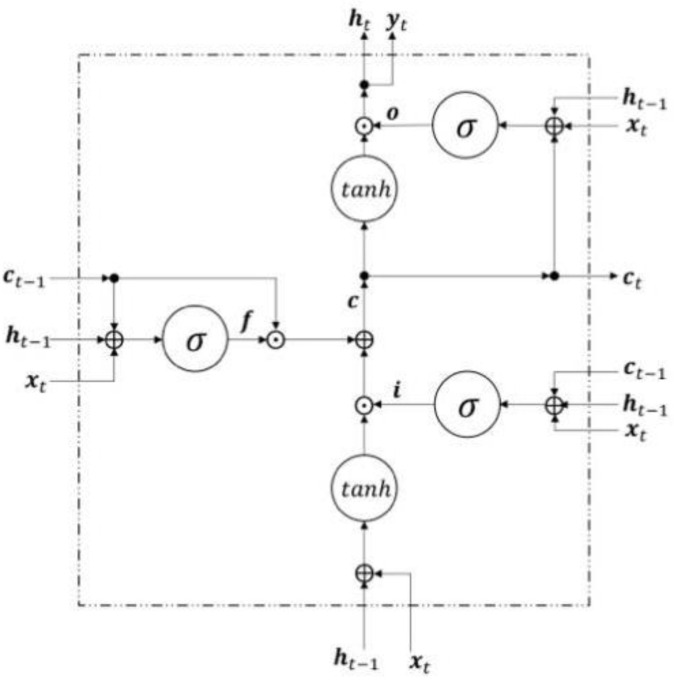
Structure of LSTM neurons.

## Appendix A

The LSTM model is a variant of the RNN model. Unlike the ordinary cell structure that represents a single function, the LSTM cell (Figure A1) has three gated functions. These gate functions are used to solve the problems of RNN gradient disappearance and gradient explosion.

For a given sequence $x=\left(x_{1},x_{2}\cdots ,x_{n}\right)$, the following state is first obtained through forward calculation:

(A1) $$i_{t}=\sigma \left(W_{xi}x_{t}+W_{hi}h_{t-1}+W_{ci}c_{t-1}+b_{i}\right)$$

(A2) $$f_{t}=\sigma \left(W_{xf}x_{t}+W_{hf}h_{t-1}+W_{cf}c_{t-1}+b_{f}\right)$$

(A3) $$o_{t}=\sigma \left(W_{xo}x_{t}+W_{ho}h_{t-1}+W_{co}c_{t}+b_{o}\right)$$

(A4) $$c_{t}=f_{t}c_{t-1}+i_{t}\tan h\left(W_{xc}h_{t-1}+W_{hc}c_{t-1}+b_{c}\right)$$

(A5) $$h_{t}=o_{t}\tan h\left(c_{t}\right)$$

Among them, i , f and o represent input gate, forget gate and output gate; c represents the cell state; and h is the hidden layer sequence, which can also be understood as the output sequence corresponding to the current input. W and b represent the weight matrix and bias term, respectively, and σ and tanh are the activation function sigmoid and hyperbolic tangent function, respectively.

After the forward calculation, LSTM starts back propagation training. First, the error of each cell is calculated backward, and then the gradient of each weight is calculated according to the error, and finally, the weight is updated according to the gradient application optimization algorithm.

## Appendix B

According to the Euclidean distance formula, the distance between the anchor and the tag can be calculated. $d_{i}$ represents the ranging distance between the tag and the i-th base station, $p_{i}$ represents the position of the i-th base station and ${\hat{p}}_{t}$ is the position of the tag:

(A6) $$d_{i}=||{\hat{p}}_{t}-p_{i}||$$

Use *θ* to represent the least-squares estimator that makes the signal model *s*[*n*] closest to the observed data *x*[*n*]. A mathematical expression can be interpreted as the least-squares estimator that makes *J(θ)* obtain the minimum value of *θ*.

(A7) $$J\left(\theta \right)\;=\;\overset{N-1}{\underset{n=0}{\sum }}{\left(x\left[n\right]\;-\;s\left[n\right]\right)}^{2}$$

In two-dimensional positioning, the Euclidean distance formula between the base station and the tag to be measured can be expressed as

(A8) $$d_{i}\;=\;\sqrt{{\left(x_{i}\;-\;x\right)}^{2}+{\left(y_{i}\;-\;y\right)}^{2}}$$

where $d_{i}$ is the distance between the tag to be measured and the *i*-th base station as measured by the UWB sensor, $x_{i}$ and $y_{i}$ are the coordinates of the i-th base station and *x* and *y* are the spatial coordinates of the tag to be measured. Expand (A8) to get

(A9) $$-2x_{i}x\;-\;2y_{i}y\;+x^{2}\;+\;y^{2}\;=\;{\hat{d_{i}}}^{2}\;-\;x_{i}^{2}\;-y_{i}^{2}$$

The following assumptions are made about the scalar parameters, where *h*[*n*] is a known sequence

(A10) s[n] = θh[n]

so, the least square error expression can be written as

(A11) $$J\left(\theta \right)\;=\;\overset{N-1}{\underset{n=0}{\sum }}{\left(x\left[n\right]\;-\;\theta h\left[n\right]\right)}^{2}$$

Write (A10) in the form of a matrix. *H* is a known *N* × *p* observation matrix and satisfies *p* full rank.

(A12) s = Hθ

The least squares estimator can be obtained by minimizing (A11), expressed as (A13)

(A13) $$J\left(\theta \right)=\overset{N-1}{\underset{n=0}{\sum }}{\left(x\left[n\right]-\theta h\left[n\right]\right)}^{2}\;=\;{\left(x\;-\;H\theta \right)}^{T}\left(x\;-\;H\theta \right)$$

(A14) $$\hat{\theta }\;=\;{\left(H^{T}H\right)}^{-1}H^{T}x$$

Introduce a new variable R to convert a nonlinear equation into a linear equation

(A15) $$R\;=\;x^{2}\;+\;y^{2}$$

Define the estimated location as

(A16) $$\hat{\theta }\;=\;{\left[x\;y\;R\right]}^{T}$$

Write the system of equations in matrix form

(A17) $$H\;=\left[\begin{array}{ccc} -2x_{1} & -2y_{1} & 1\\ \begin{array}{c} -2x_{2}\\ \vdots \end{array} & \begin{array}{c} -2y_{2}\\ \vdots \end{array} & \begin{array}{c} 1\\ \vdots \end{array}\\ -2x_{N} & -2y_{N} & 1 \end{array}\right]$$

(A18) $$x\;=\;\left[\begin{array}{c} {\hat{d}}_{1}^{2}-\;x_{1\;}^{2}-\;y_{1}^{2}\\ \begin{array}{c} {\hat{d}}_{2}^{2}-\;x_{2\;}^{2}-\;y_{2}^{2}\\ \vdots \end{array}\\ {\hat{d}}_{N}^{2}-\;x_{N\;}^{2}-\;y_{N}^{2} \end{array}\right]$$

The above formula predefines a linear equation system in normal form, in which the label position can be calculated by the least square estimator defined in (A16).

## Appendix C

For the ordinary least square method described in Part B, it implies the assumption that the variance of the ranging error is constant. However, in actual positioning, the NLOS condition usually has a larger ranging error. Weighting individual positions according to predicted ranging errors helps to obtain more reliable position estimates.

Introduce weighting matrix *W*,

(A19) $$W\;=\;\left[\begin{array}{ccc} \begin{array}{c} w_{1}\\ 0 \end{array} & \begin{array}{cc} \begin{array}{c} 0\\ w_{2} \end{array} & \begin{array}{c} \ldots \\ \ldots \end{array} \end{array} & \begin{array}{c} 0\\ 0 \end{array}\\ \vdots & \;\begin{array}{cc} \vdots & \;\ddots \end{array} & \vdots \\ 0 & \;\begin{array}{cc} 0 & \;\ldots \end{array} & w_{n} \end{array}\right]$$

than the formula (A7) for estimating the least squares error can be updated as

(A20) $$J\left(\theta \right)=\overset{N-1}{\underset{n=0}{\sum }}w_{i}{\left(x\left[n\right]-\theta h\left[n\right]\right)}^{2}$$

(A21) $$=\;{\left(x\;-\;H\theta \right)}^{T}W\left(x\;-\;H\theta \right)$$

Calculate weights $w_{i}$ for individual range results using range distance $d_{i}$ and range error $\epsilon_{i}$

(A22) $$w_{i}\;=\;\frac{1}{{\left(d_{i}\epsilon_{i}\right)}^{2}}$$

The estimated $\;\hat{\theta }$ for the position of tag can be expressed in terms of weighted least squares as

(A23) $${\hat{\theta }}_{WLS}\;=\;{\left(H^{T}WH\right)}^{-1}H^{T}Wx$$
